# Erratum to “MiNuGAN: Dual segmentation of mitoses and nuclei using conditional GANs on multi-center breast H&E images” [Journal of Pathology Informatics Volume 13, 2022, 100002]

**DOI:** 10.1016/j.jpi.2025.100426

**Published:** 2025-04-29

**Authors:** Salar Razavi, Fariba D. Khameneh, Hana Nouri, Dimitrios Androutsos, Susan J. Done, April Khademi

**Affiliations:** a Electrical, Computer and Biomedical Engineering, Ryerson University, Toronto, Ontario, Canada; b Laboratory Medicine Program, University Health Network, Toronto, Ontario, Canada

The publisher regrets that the Declaration of Competing Interests was missing in the published article. The statement should read as follows:

## Declaration of competing interest

The authors declare the following financial interests/personal relationships which may be considered as potential competing interests:

Salar Razavi reports financial support was provided by Ryerson University Department of Electrical Computer and Biomedical Engineering. Salar Razavi reports a relationship with Ryerson University Department of Electrical Computer and Biomedical Engineering that includes: employment. If there are other authors, they declare that they have no known competing financial interests or personal relationships that could have appeared to influence the work reported in this paper.

The publisher would like to apologise for any inconvenience caused.

