# Supplementary material for: MiNuGAN: Dual Segmentation of Mitoses and Nuclei Using Conditional GANs on Multi-center Breast H&E Images
Source: J Pathol Inform. 2022 Jan 20;13:100002. doi: 10.1016/j.jpi.2022.100002 (PMC8860738; doi:10.1016/j.jpi.2022.100002)
Supplement: Supplementary file 1 — Supplementary material [file mmc1.pdf]

Hello,

The paper is sent to the JPI on September 12, then revised on November 19 and accepted on November 19th.

The APC is already paid with an invoice number of 2396.

The manuscript number is jpi_63_21.

All the required files of the paper is also attached here.

1–19

MiNuGAN - Dual Segmentation of Mitoses and Nuclei using Conditional GANs on Multicentre Breast H&E Images

Salar Razavi[1] SALAR.RAZAVI@RYERSON.CA
Fariba D. Khameneh[1] FDAMBANDKHAMENEH@RYERSON.CA
Hana Nouri[1] HANA.NOURI@RYERSON.CA
Dimitrios Androutsos[1] DIMITRI@RYERSON.CA
Susan Done[2] SUSAN.DONE@UHN.CA
April Khademi[1] AKHADEMI@RYERSON.CA
[1] *Electrical, Computer and Biomedical Engineering, Ryerson University, Toronto, Ontario, Canada.*
[2] *Laboratory Medicine Program, University Health Network, Toronto, Ontario, Canada.*

Abstract

Breast cancer is the second most commonly diagnosed type of cancer among women as of 2021. Grading of histopathological images is used to guide breast cancer treatment decisions and a critical component of this is a mitotic score, which is related to tumor aggressiveness. Manual mitosis counting is an extremely tedious manual task, but automated approaches can be used to overcome inefficiency and subjectivity. In this paper, we propose an automatic mitosis and nuclear segmentation method for a diverse set of H&E breast cancer pathology images. The method is based on a conditional generative adversarial network (cGAN) to segment both mitoses and nuclei at the same time. Architecture optimizations are investigated, including hyperparameters and the addition of a focal loss. The accuracy of the proposed method is investigated using images from multiple centres and scanners, including TUPAC16, ICPR14 and ICPR12 datasets. In TUPAC16, we use 618 carefully annotated images of size 256x256 scanned at 40×. TUPAC16 is used to train the model, and segmentation performance is measured on the test set for both nuclei and mitoses. Results on 200 held-out testing images from the TUPAC16 dataset were mean DSC = 0.784 and 0.721 for nuclear and mitosis, respectively. On 202 ICPR12 images, mitosis segmentation accuracy had a mean DSC = 0.782, indicating the model generalizes well to unseen datasets. For datasets that had mitosis centroid annotations, which included 200 TUPAC16, 202 ICPR12 and 524 ICPR14, a mean F1 score of 0.854 was found indicating high mitosis detection accuracy.

Keywords: Mitosis detection, semantic segmentation, generative adversarial network, breast cancer, focal loss, computer-aided detection

1. Introduction

Mitosis is the process of cell duplication, in which one cell divides into two genetically identical daughter cells (Sebai et al., 2020). In breast tumours, the number of mitotic figures is related to tumour aggressiveness and proliferation (Van Diest et al., 2004) and is used for tumour grading and determining treatment options. Experienced pathologists determine mitotic counts through visual examination of hematoxylin and eosin (H&E) stained tissue sections by counting the number of mitotic figures in ten high-power fields (HPF). Due to large variations in mitosis appearance, the labourious and subjective nature of the task, as

© S. Razavi, F.D. Khameneh, H. Nouri, D. Androutsos, S. Done & A. Khademi.

well as variability in preparation protocols (staining, scanning), manual mitosis counts have up to 25% discordance between pathologists (Elmore et al., 2015).

Computer-aided diagnostic (CAD) tools hold promise to overcome the labourious and subjective nature of mitotic grading (Mercan et al., 2019) by providing robust, consistent and efficient analysis. The public mitosis detection challenges at ICPR12 (Roux et al., 2013), AMIDA13 (Veta et al., 2015), ICPR14 (Roux) and TUPAC16 (Veta et al., 2019) have facilitated development of various artificial intelligence (AI) solutions to this problem. These methods employed a variety of tools from handcrafted features (Irshad et al., 2013), deep learning (Li et al., 2018) or a combination of both machine learning (ML) and deep learning (DL) systems (Malon and Cosatto, 2013; Mahmood et al., 2020).

Recently, DL systems for automated mitosis detection in breast cancer images have gained in popularity due to the ability of these systems to adapt to diverse data with often superior performance compared to traditional machine learning methods (Irshad et al., 2013) (Irshad et al., 2014). The DL methods employed on TUPAC16, ICPR12 and ICPR14 datasets have largely considered convolutional neural networks (CNN) to classify candidate regions (Das and Dutta, 2019) or to detect mitotic figures (Sebai et al., 2020). State-of-the-art methods apply a multi-stage methodology to locate ROI with candidate mitoses and then detect cells in mitosis in those regions (Ma et al., 2018a; Paeng et al., 2017; Sebai et al., 2020; Li et al., 2018; Li. et al., 2018; Aubreville et al., 2019; Janowczyk and Madabhushi, 2016). To improve the performance and generalization, pre-processing methods are commonly employed such as wavelet decomposition (Wan et al., 2017), color deconvolution (Tellez et al., 2018; Irshad, 2013) and stain normalization (Das and Dutta, 2019; Lafarge et al., 2017; Ma et al., 2018b). While most methods focus on obtaining mitotic counts through mitosis detection, there could be value in segmenting the mitosis as a whole, which can easily facilitate mitosis counts, and can be used to analyze properties of the mitoses themselves (such as mitotic stage). Mitosis segmentation is the focus of this work.

Generative adversarial networks (GANs) have recently been applied to translate histopathology images between domains. Rana *et. al* utilized a GAN-based CNN network to virtually stain hematoxylin and eosin (H&E) specimens. Hou *et. al* (Hou et al., 2017) synthesize histopathology images based on nuclei masks which are then used for task-specific CNN training. Jerry Wei *et. al* (Wei et al., 2019) presented an image translation methodology to generate augmented data to mitigate data imbalance issues in colorectal histopathology images. Bentaib *et. al* uses a discriminator with stain normalization that transfers stains across different datasets with various staining appearance. Tellez *et. al* (Tellez et al., 2019) use an unsupervised image-to-image translation for color normalization and augmentation and the effects of these preprocessing steps are evaluated on different classification tasks. In (Quiros et al., 2019), PathologyGAN is proposed to segment nuclei and learn pathological features within cancer tissue images to correlate breast cancer disease with molecular information. Mahmood *et. al* (Mahmood et al., 2019) use a conditional GAN to generate H&E training images with labels and both synthetic and real images are utilized to train an unpaired GAN framework to segment nuclei.

Inspired by the challenges of mitosis segmentation and previous works in image translation using GANs, this paper proposes the use of conditional generative adversarial networks (cGANs) to segment both mitoses and nuclei at the same time. cGANs may hold potential by constraining solutions to be more ”realistic” through discriminators which could miti-

gate inconsistency and generalization challenges. Although the main application is mitosis segmentation and detection, dual segmentation of nuclei serves two purposes. Firstly, providing the classifier with annotations of non-dividing nuclei may help the system learn the mitosis class more effectively. Secondly, segmenting nuclei can help to facilitate automated nuclear grading.

The cGAN model utilized in this work consists of a conditional encoder-decoder network for a generator which offers a coarse to fine representation of imaging data, and a multi-scale discriminator network that uses both local and global scales to differentiate between real and fake images. The result is a translated image that represents the segmented mitoses and nuclei. Ground truth delineations for nuclei and mitoses provide conditional labels for the generator and a focal loss is investigated to emphasize the mitosis (imbalanced) class. Adversarial and feature matching losses from multiple scales are utilized. Training on multiple scales enables the architecture to generate images with finer details.

Mitosis and nuclear annotations were developed for 618 images from the TUPAC16 dataset (3 centres) where 418 were used to train and validate the segmentation model. This dataset was acquired from three different centres and has wide colour variability. Segmentation accuracy for both nuclei and mitoses is measured using the DSC through five fold cross validation, and is compared to baseline U-Net and U-Net with focal loss. To examine mitosis detection in unseen datasets, the model was tested on images from ICPR12 (202), ICPR14 (524) and heldout TUPAC16 (200). In multicentre breast cancer pathology images, data variability can arise from different staining protocols, scanner vendors and patients with different tissue morphology which can create generalization challenges for deep learning methods. Therefore, experimenting with large and diverse datasets is needed to optimize such tools for clinical translation.

2. Methods

2.1. Dataset

In this work, we consider three open source datasets for mitosis detection and segmentation: TUPAC16, ICPR12 and ICPR14. From each dataset, random patches of size 256x256 were extracted from the HPF from each dataset such that patches had at least one mitosis that was not on the boundary. TUPAC16 has a total of 656 HPF for 73 patients, scanned at three different centres. Annotations for both nuclei and mitoses were generated for TUPAC16 since it contains more variability (3 centres with wide stain variations). Annotations were generated using Pathcore Sedeen (Martel et al., 2017) by a biomedical student trained by a breast pathologist. Pixel-level annotations were completed for 48 of the 73 patients resulting in 418 patches for training and validation. This dataset is used to fine-tune the segmentation model and to determine validation performance over all 418 patches through five fold cross validation. Other model comparisons also use this dataset to compare segmentation performance. Example annotations are shown in Figure 1.

During testing, patches from 25 patients from TUPAC16 are used to determine held-out mitosis and nuclear segmentation performance. Images from these patients are not used during training. The number of patches from the 25 patients for testing was 200. In the ICPR12 dataset, there are images with pixel-level annotations for mitosis from 5 patients, acquired using two scanners (Aperio and Hamamatsu) for a total of 70 HPF. In this work,

Table 1: Experimental datasets.

Dataset	Patches	Centres	Scanners	Mitoses	Labels
TUPAC16	618	3	Leica, Aperio	730	Pixel-level (mitosis, nuclear), centroid (mitosis)
ICPR14	524	1	Aperio, Hamamatsu	550	Centroid (mitosis)
ICPR12	202	1	Aperio, Hamamatsu	226	Pixel-level (mitosis), centroid (mitosis)

202 test images of size 256x256 were sampled from the HPF to create the ICPR12 test set. In ICPR14, there are 11 HPF scanned with Aperio and Hamamatsu scanners and 524 test images of size 256x256 were sampled from all HPF. In this dataset, only mitosis centroid annotations are available. ICPR12 and ICPR14 datasets are used to measure generalization performance. The total number of patches generated for each dataset is described in Table 1. All the images are scanned at 40x magnifications.

2.2. MiNuGAN - Dual Mitosis and Nuclear Segmentation

The proposed cGAN architecture called MiNuGAN for dual mitosis and nuclear segmentation is adapted from the original pix2pixHD cGAN architecture (Wang et al., 2018) and is shown in Figure 2. An encoder-decoder architecture with ResNet (He et al., 2016) blocks are used for the generator network, consisting of 5 convolutional layers and 9 residual blocks in the encoding arm, 4 convolutional upsampling layers in the decoder, followed by 3 residual blocks after deconvolution and 2 more convolution layers. The output is a generated segmentation mask for both mitosis and nuclear classes. After each convolution layer, instance normalization (Pan et al., 2018) is employed, followed by rectified linear unit (ReLU) activation. The coarse-to-fine generator encodes features from multiple scales, which permits for different sized-details to be detected. Skip connections are added between the local enhancer (G_1) and global (G_2) enhancer which aims to maintain and transfer global information from G_1 to G_2. G_1 and G_2 generators are jointly trained to generate masks from input images and the inputs are 128×128 and 256×256 in size, respectively. The last convolution layer in the architecture is followed by Tanh. The discriminator in Figure 2 predicts whether the generated segmentation masks are real or fake. The discriminators use images of size 256x256, 128x128 and 64x64 for scales 1, 2 and 3. The baseline discriminator has three multi-scale discriminators (D_1, D_2, D_3) which use images of different resolutions to predict image realism based on multiple resolutions. For each scale, the discriminator adopts the PatchGAN (Isola et al., 2017) architecture with four convolutional layers, followed by a sigmoid output layer for classification. The multi-scale PatchGAN discriminator classifies patches of various resolutions. The multi-scale D_1, D_2 and D_3 discriminators are trained simultaneously to distinguish the generated segmentation masks from ground truth images.

In this work a focal loss (Lin et al., 2017) is implemented in the generator and deployed after the last hidden layer to improve mitosis and nuclear segmentation performance. Unlike general classification tasks that assume an equal occurrence from all of the classes, medical applications suffer small sample sizes and resulting class imbalances. In mitosis segmentation there is a heavy imbalance between the classes (there are much more nuclei

than mitosis pixels) which can hamper the detection of mitotic cells (Veta et al., 2015). Training a network on imbalanced datasets can make the network biased towards learning more representations of the data-dominated class (i.e. nuclei) while other classes (i.e. mitosis) will receive less importance. A focal loss can be used to reduce the contribution of the more commonly occurring nuclear pixels and focus the classifier on the harder to predict (mitosis) pixels and is incorporated into the total loss function for cGANs in this work. The total loss function for cGANs is a combination of the adversarial loss L_{GAN} and the feature matching (FM) loss L_{FM} as in

$$\min_{G} \max_{D_1,D_2} \sum_{k=1,2} L(D_k, G) = \min_{G} \max_{D_1,D_2} \sum_{k=1,2} L_{GAN}(D_k, G) + \lambda \sum_{k=1,2} L_{FM}(D_k, G) \tag{1}$$

where λ balances the influence of the feature matching loss L_{FM} with the adversarial loss L_{GAN} to prevent overfitting to specific distributions. The feature matching loss considers dissimilarities between feature representations in local and global levels. The adversarial loss tries to maximize the probability of real images and minimize the probability of fake inputs to the discriminator. In this work, L_{GAN} is computed by considering a focal loss parameter γ that reduces or increases the relative loss for well-classified or poorly-classified pixels, as in

$$L_{GAN}(D_k, G)_{FL} = \mathbb{E}[(1 - D)^{\gamma} \log(D)] + \mathbb{E}[D^{\gamma} \log(1 - D)]. \tag{2}$$

The output of the model is a mutliclass image with pixel-level labels for each of the predicted mitosis and nuclear objects. To measure segmentation performance, pixel-level predictions are compared to ground truth delineations on a per-pixel basis. For mitosis detection, the mitosis segmentation masks are used to automatically determine the centroids of each mitosis object.

2.3. Metrics

We evaluate segmentation performance of the cGAN model using the dice similarity coefficient (DSC) which compares automated segmentations to manual delineations for the mitosis and nuclear classes separately. To measure mitosis detection performance on datasets with ground truth mitosis centroids, the F1-score is utilized. Precision and sensitivity are determined by the number of correctly predicted mitosis centroids within a 25 pixel-size window centered on the ground truth centroid. This corresponds to a distance of 7 μm and was chosen since it is the average of the mitosis competitions.

3. Results and Discussion

All models are developed using TUPAC16 data with pixel-level annotations of nuclei and mitoses (334/84 training/validation split and five fold cross validation). Average segmentation performance is reported for all 418 patches for nuclei and mitoses separately. Model fine-tuning is done using the detailed annotation dataset and the final dual nuclear and mitosis segmentation model termed "MiNuGAN" is carried forward for further analysis. To measure generalization performance of the proposed system, two held out test sets with pixel-level annotations were used. There were 200 patches from TUPAC16 that had mitosis

and nuclear annotations, and 202 patches from ICPR12 with mitosis delineations. The segmentation results of MiNuGAN are compared to baseline models, such as U-Net and U-Net with a focal loss, which all were trained using the same five fold cross validation datasets.

In addition to measuring segmentation performance, additional experiments were conducted to measure mitosis detection accuracy through the F1 scores. These are reported all held out datasets for mitosis centroid annotations, which includes: ICPR12 (202 patches), ICPR14 (524 patches) and for the held out TUPAC16 set (200 patches).

All models are trained on Nvidia 2080TI GPU with 11 GB memory. The training images consist of original images along with RGB label maps (with each colour representing either the nuclei, boundary or background class). All images are of size 256x256.

3.1. Mitosis and Nuclear Segmentation

Performance of the proposed mitosis and nuclear segmentation model is reported in this section. In total, 418 patches (334 training/84 validation, five fold cross validation) from TUPAC16 are used for training and model fine-tuning without any preprocessing such as stain normalization. The dataset is split in a 5-fold stratified fashion (mixing the validation and training sets) and average performance on the validation data over five folds is reported. Each model is trained for 100 epochs with a learning rate of 0.5. The baseline pix2pixHD model has three multi-scale discriminators, 9 residual blocks in the generator and uses $\lambda = 5$. In addition to these parameters, we also investigated a focal loss with $\gamma = 0, 0.1, 1, 2, 5$ to increase the importance of mitosis when classifying pixels. We varied the number of discriminators, residual blocks, λ and the focal loss and report some of the top performing configurations for mitosis and nuclear segmentation. In total, this resulted in ten configurations as outlined in Table 2. The average DSC over both mitosis and nuclear classes in the validation set, for five folds for each configuration is shown in the last column of Table 2, and graphically in Figure 3 and Figure 4. The highest DSC for the mitosis class is obtained for configurations that use a focal loss and baseline parameters: 3 multiscale discriminators, 9 residual blocks and $\gamma = 2$. This configuration also results in the lowest coefficient of variation, indicating that this model segments mitoses with the highest consistency. Visual results for the top performing method (baseline + focal loss) and the same architecture without focal loss are shown Figure 6. Without focal loss there are lots of small false positive mitoses that would decrease mitosis detection accuracy. Predictions based on a focal loss have more accurately segmented the mitotic figures. When considering the DSC for the nuclear and mitosis class separately, it is apparent that lower performance is generally obtained for the cells in mitosis. This could be a result of the complex nature and variable appearance of mitotic cells and could also be due to a relative class imbalance. However, as shown in Figure 6 the focal loss improves the imbalance issue and segments mitoses with improved accuracy. Furthermore, in Figure 3 and Figure 4, using fewer residual blocks in the generator decreases overall DSC performance and 9 ResNet blocks (as per baseline Pix2pixHD) seems appropriate for this task. Results also show that increasing the number of the discriminators from 2 to 3 increases the DSC, which may indicate that mitotic cells are better detected at lower resolutions. For the remainder of this work, we use the baseline cGAN (3 multiscale discriminators, 9 residual blocks, and $\gamma = 2$) with focal

loss and call this configuration MiNuGAN.

Using the finalized model MiNuGAN just described, the mitosis segmentation testing performance on the held out TUPAC16 and ICPR12 is computed. In total, there were 402 patches, and the results are summarized in Figure 5. The mean mitosis and nuclear segmentation performance on TUPAC16 were: 72.1% and 78.4%, respectively. On ICPR12, the mean mitosis segmentation accuracy was 78.2%, which is slightly higher than the TUPAC16 dataset (on which the model was trained). This may be due to a number of factors. Firstly, the TUPAC16 dataset has large variability and may be allowing the model to learn more diverse features which permits for better generalization to unseen data. Second, the ICPR12 dataset is of relatively high contrast, which may permit for better mitosis segmentation. Third, the discriminator function allows the classifier to learn realistic mitosis patterns. The high segmentation performance across both datasets is a good indication that these features are beneficial for generalization to new data.

Table 2: cGAN configurations and average DSC of mitotic and nuclear classes

Configuration	Discrim	Resblocks	Lambda	DSC
1	2	9	0	0.739±0.208
2	2	9	0.1	0.742±0.200
3	2	9	1	0.741±0.196
4	2	9	2	0.747±0.194
5	2	9	5	0.738±0.208
6	3	9	0	0.747±0.189
7	3	9	0.1	0.747±0.199
8	3	9	1	0.737±0.210
9	3	9	2	**0.758±0.192**
10	3	9	5	0.752±0.185

To compare the performance of the MiNuGAN to baseline systems, we compared segmentation results over the validation set with U-Net and U-Net with a focal loss. These systems were trained for 100 epochs using Adam optimizer, 0.001 learning rate, batch size of 16, four layers and with/without focal loss. The same training and validation datasets were used in a five fold cross validation manner. Figure 7 shows the DSC distribution and Figure 8 shows the mean DSC and the DSC coefficient of variation (CoV) of both U-Net models versus MiNuGAN. The mean DSC segmentation performance is lowest for U-NET without focal loss, which is slightly improved when a focal loss is used. The highest mean DSC performance is achieved by MiNuGAN for both nuclear and mitosis datasets. Additionally, the coefficient of variation is significantly lower for the proposed method, indicating results are more consistent in this multicentre dataset. Visual results are shown in Figure 9. U-Net misses mitoses and also generates more false positives, even with focal loss. In contrast, the proposed method has less false positives and negatives, and the segmentation masks are smoother, which may be attributed to the multiscale discriminator which forces the output segmentation masks to be more realistic (i.e. similar to ground truths).

3.2. Mitosis Detection Performance using MiNuGAN

Using the baseline cGAN system with focal loss (MiNuGAN), the detection performance of the proposed method is evaluated on images from multiple datasets and scanners (heldout TUPAC16 samples: 200 images, ICPR12: 202 images and ICPR14: 524 images). As the

model predicts both nuclei and mitoses on a per-pixel basis, only mitosis labels are retained for detection. Morphological processing is applied to the mitosis masks (small erosion, hole closing) and the centroid of every object is determined. The ground truth centroid is compared to the predicted centroids through the F1. See Table 3 for the average F1 performance for each dataset. The average F1 over all three datasets was found to be 0.854±0.268 and considering that over 80% of the data comes from sources that were not seen by the classifier during training this is a very promising result. F1-scores on ICPR12 and ICPR14 were lower compared with the TUPAC16 dataset (roughly 10% performance drop). See Fig. 10 for example mitosis detection results for MiNuGAN over all three datasets. The variation between dataset distributions (colour, stain, patient, scanner) could be the cause for some of the challenges in ICPR12 and ICPR14, although over all the model is performing well. The last column in Figure 10 shows example segmentations in patches with no mitoses. As can be seen, only nuclei were detected in these images. In future works, we will apply this method on whole-slide images and determine the effect of negative patches on performance. We may consider an RCNN to find candidate regions first, and then use MiNuGAN to segment and detect mitoses from those patches. To improve generalization, we will investigate colour normalization as a means to mitigate stain and colour variability in multicentre datasets.

Table 3: Mitosis detection performance on different datasets and scanners.

	**Dataset**			**Scanner**	
	TUPAC16	ICPR14	ICPR12	Aperio	Hamamatsu
**F1-score**	0.872	0.847	0.851	0.832	0.881

4. Conclusion

This work proposes a cGAN-based model called MiNuGAN that segments both mitoses and nuclei at the same time from H&E stained breast cancer images. It is based on the baseline pix2pixHD model, except a focal loss has been added to bring attention to the imbalanced mitosis class. Testing results show that segmentation performance is high for both mitoses and nuclei in TUPAC16 with mean DSC = 0.721 and 0.784 for mitoses and nuclei, respectively, over 200 image patches and mean DSC = 0.782 for 202 ICPR12 images. Results show the proposed method generates more realistic and accurate segmentation maps compared to baseline U-Net models, even U-Net with focal loss, indicating the discriminator forces continuity in the segmentation mask that is beneficial for segmentation. Using the mitosis segmentations, mitosis centroids were detected and compared to datasets with centroid annotaitons. The F1 scores were computed over 3 multicentre H&E datasets (TUPAC16, ICPR12, ICPR14) and an average F1 score of 0.854 was achieved. Ablation studies show the effectiveness of the focal loss to increase the performance of mitosis segmentation and detection. In the future, stain normalization will be considered, as well as augmentation to see if performance across scanners and centres can be improved. In this work, we focused mainly on mitosis segmentation and detection in breast cancer H&E images. In the future, it is possible to evaluate MiNuGAN on different tissue types and datasets.

References

Marc Aubreville, Christof A Bertram, Robert Klopfleisch, and Andreas Maier. Augmented mitotic cell count using field of interest proposal. In *Bildverarbeitung für die Medizin 2019*, pages 321–326. Springer, 2019.

Dev Kumar Das and Pranab Kumar Dutta. Efficient automated detection of mitotic cells from breast histological images using deep convolution neutral network with wavelet decomposed patches. *Computers in biology and medicine*, 104:29–42, 2019.

Joann G Elmore, Gary M Longton, Patricia A Carney, Berta M Geller, Tracy Onega, Anna NA Tosteson, Heidi D Nelson, Margaret S Pepe, Kimberly H Allison, Stuart J Schnitt, et al. Diagnostic concordance among pathologists interpreting breast biopsy specimens. *Jama*, 313(11):1122–1132, 2015.

Kaiming He, Xiangyu Zhang, Shaoqing Ren, and Jian Sun. Deep residual learning for image recognition. In *Proceedings of the IEEE conference on computer vision and pattern recognition*, pages 770–778, 2016.

Le Hou, Ayush Agarwal, Dimitris Samaras, Tahsin M Kurc, Rajarsi R Gupta, and Joel H Saltz. Unsupervised histopathology image synthesis. *arXiv preprint arXiv:1712.05021*, 2017.

Humayun Irshad. Automated mitosis detection in histopathology using morphological and multi-channel statistics features. *Journal of pathology informatics*, 4, 2013.

Humayun Irshad, Ludovic Roux, and Daniel Racoceanu. Multi-channels statistical and morphological features based mitosis detection in breast cancer histopathology. In *2013 35th Annual International Conference of the IEEE Engineering in Medicine and Biology Society (EMBC)*, pages 6091–6094. IEEE, 2013.

Humayun Irshad, Alexandre Gouaillard, Ludovic Roux, and Daniel Racoceanu. Multispectral band selection and spatial characterization: Application to mitosis detection in breast cancer histopathology. *Computerized Medical Imaging and Graphics*, 38(5):390–402, 2014.

Phillip Isola, Jun-Yan Zhu, Tinghui Zhou, and Alexei A Efros. Image-to-image translation with conditional adversarial networks. In *Proceedings of the IEEE conference on computer vision and pattern recognition*, pages 1125–1134, 2017.

Andrew Janowczyk and Anant Madabhushi. Deep learning for digital pathology image analysis: A comprehensive tutorial with selected use cases. *Journal of pathology informatics*, 7, 2016.

Maxime W Lafarge, Josien PW Pluim, Koen AJ Eppenhof, Pim Moeskops, and Mitko Veta. Domain-adversarial neural networks to address the appearance variability of histopathology images. In *Deep Learning in Medical Image Analysis and Multimodal Learning for Clinical Decision Support*, pages 83–91. Springer, 2017.

Chao Li, Xinggang Wang, Wenyu Liu, and Longin Jan Latecki. Deepmitosis: Mitosis detection via deep detection, verification and segmentation networks. *Medical image analysis*, 45:121–133, 2018.

Yuguang Li., Ezgi Mercan., Stevan Knezevitch., Joann G. Elmore., and Linda G. Shapiro. Efficient and accurate mitosis detection - a lightweight rcnn approach. In *Proceedings of the 7th International Conference on Pattern Recognition Applications and Methods - Volume 1: ICPRAM,*, pages 69–77. INSTICC, SciTePress, 2018. ISBN 978-989-758-276-9. doi: 10.5220/0006550700690077.

Tsung-Yi Lin, Priya Goyal, Ross Girshick, Kaiming He, and Piotr Dollár. Focal loss for dense object detection. In *Proceedings of the IEEE international conference on computer vision*, pages 2980–2988, 2017.

Minglin Ma, Yinghuan Shi, Wenbin Li, Yang Gao, and Jun Xu. A novel two-stage deep method for mitosis detection in breast cancer histology images. In *2018 24th International Conference on Pattern Recognition (ICPR)*, pages 3892–3897. IEEE, 2018a.

Yanbo Ma, Jiarui Sun, Qiuhao Zhou, Kaili Cheng, Xuesong Chen, and Yong Zhao. Chs-net: A cascaded neural network with semi-focal loss for mitosis detection. In *Asian Conference on Machine Learning*, pages 161–175, 2018b.

Faisal Mahmood, Daniel Borders, Richard Chen, Gregory N McKay, Kevan J Salimian, Alexander Baras, and Nicholas J Durr. Deep adversarial training for multi-organ nuclei segmentation in histopathology images. *IEEE transactions on medical imaging*, 2019.

Tahir Mahmood, Muhammad Arsalan, Muhammad Owais, Min Beom Lee, and Kang Ryoung Park. Artificial intelligence-based mitosis detection in breast cancer histopathology images using faster r-cnn and deep cnns. *Journal of Clinical Medicine*, 9(3):749, 2020.

Christopher D Malon and Eric Cosatto. Classification of mitotic figures with convolutional neural networks and seeded blob features. *Journal of pathology informatics*, 4, 2013.

Anne L Martel, Dan Hosseinzadeh, Caglar Senaras, Yu Zhou, Azadeh Yazdanpanah, Rushin Shojaii, Emily S Patterson, Anant Madabhushi, and Metin N Gurcan. An image analysis resource for cancer research: Piip—pathology image informatics platform for visualization, analysis, and management. *Cancer research*, 77(21):e83–e86, 2017.

Ezgi Mercan, Sachin Mehta, Jamen Bartlett, Linda G Shapiro, Donald L Weaver, and Joann G Elmore. Assessment of machine learning of breast pathology structures for automated differentiation of breast cancer and high-risk proliferative lesions. *JAMA network open*, 2(8):e198777–e198777, 2019.

Kyunghyun Paeng, Sangheum Hwang, Sunggyun Park, and Minsoo Kim. A unified framework for tumor proliferation score prediction in breast histopathology. In *Deep Learning in Medical Image Analysis and Multimodal Learning for Clinical Decision Support*, pages 231–239. Springer, 2017.

Xingang Pan, Ping Luo, Jianping Shi, and Xiaoou Tang. Two at once: Enhancing learning and generalization capacities via ibn-net. In *Proceedings of the European Conference on Computer Vision (ECCV)*, pages 464–479, 2018.

Adalberto Claudio Quiros, Roderick Murray-Smith, and Ke Yuan. Pathology gan: learning deep representations of cancer tissue. *arXiv preprint arXiv:1907.02644*, 2019.

Roux. MITOS-ATYPIA-14. https://mitos-atypia-14.grand-challenge.org/.

Ludovic Roux, Daniel Racoceanu, Nicolas Loménie, Maria Kulikova, Humayun Irshad, Jacques Klossa, Frédérique Capron, Catherine Genestie, Gilles Le Naour, and Metin N Gurcan. Mitosis detection in breast cancer histological images an icpr 2012 contest. *Journal of pathology informatics*, 4, 2013.

Meriem Sebai, Xinggang Wang, and Tianjiang Wang. Maskmitosis: a deep learning framework for fully supervised, weakly supervised, and unsupervised mitosis detection in histopathology images. *Medical & Biological Engineering & Computing*, 2020.

David Tellez, Maschenka Balkenhol, Irene Otte-Höller, Rob van de Loo, Rob Vogels, Peter Bult, Carla Wauters, Willem Vreuls, Suzanne Mol, Nico Karssemeijer, et al. Whole-slide mitosis detection in h&e breast histology using phh3 as a reference to train distilled stain-invariant convolutional networks. *IEEE transactions on medical imaging*, 37(9): 2126–2136, 2018.

David Tellez, Geert Litjens, Péter Bándi, Wouter Bulten, John-Melle Bokhorst, Francesco Ciompi, and Jeroen van der Laak. Quantifying the effects of data augmentation and stain color normalization in convolutional neural networks for computational pathology. *Medical image analysis*, 58:101544, 2019.

Paul J Van Diest, Elsken van der Wall, and Jan PA Baak. Prognostic value of proliferation in invasive breast cancer: a review. *Journal of clinical pathology*, 57(7):675–681, 2004.

Mitko Veta, Paul J Van Diest, Stefan M Willems, Haibo Wang, Anant Madabhushi, Angel Cruz-Roa, Fabio Gonzalez, Anders BL Larsen, Jacob S Vestergaard, Anders B Dahl, et al. Assessment of algorithms for mitosis detection in breast cancer histopathology images. *Medical image analysis*, 20(1):237–248, 2015.

Mitko Veta, Yujing J Heng, Nikolas Stathonikos, Babak Ehteshami Bejnordi, Francisco Beca, Thomas Wollmann, Karl Rohr, Manan A Shah, Dayong Wang, Mikael Rousson, et al. Predicting breast tumor proliferation from whole-slide images: the tupac16 challenge. *Medical image analysis*, 54:111–121, 2019.

Tao Wan, Wanshu Zhang, Min Zhu, Jianhui Chen, Alin Achim, and Zengchang Qin. Automated mitosis detection in histopathology based on non-gaussian modeling of complex wavelet coefficients. *Neurocomputing*, 237:291–303, 2017.

Ting-Chun Wang, Ming-Yu Liu, Jun-Yan Zhu, Andrew Tao, Jan Kautz, and Bryan Catanzaro. High-resolution image synthesis and semantic manipulation with conditional gans. In *Proceedings of the IEEE conference on computer vision and pattern recognition*, pages 8798–8807, 2018.

Jerry Wei, Arief Suriawinata, Louis Vaickus, Bing Ren, Xiaoying Liu, Jason Wei, and Saeed Hassanpour. Generative image translation for data augmentation in colorectal histopathology images. *arXiv preprint arXiv:1910.05827*, 2019.

Appendix A.1. Additional Figures

This appendix includes additional figures that are referenced in the manuscript.

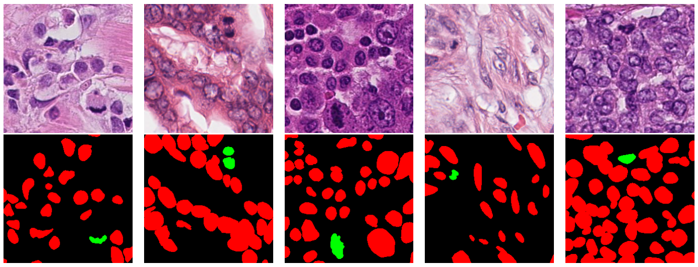

Figure 1: Mitosis (green) and nuclear (red) annotations from TUPAC16.

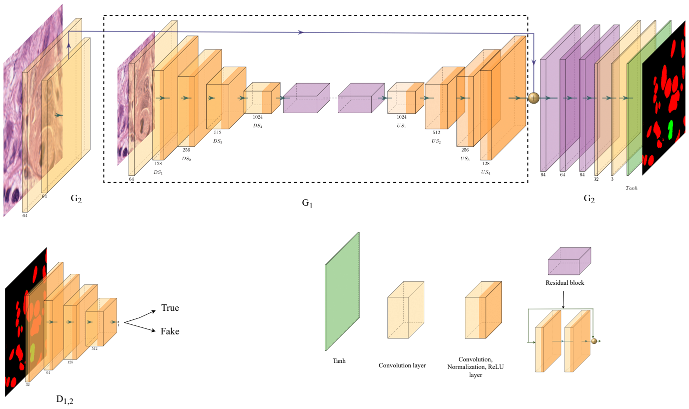

Figure 2: MiNuGAN for dual mitosis and nuclear segmentation in H&E breast cancer images.

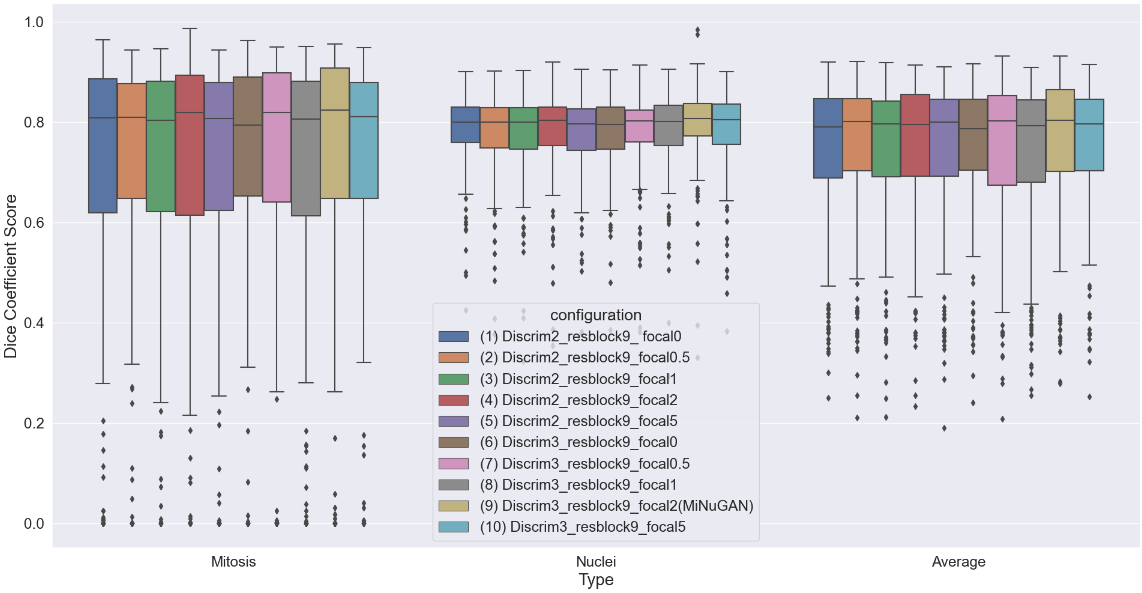

Figure 3: DSC distributions for cGAN configurations in Table 2.

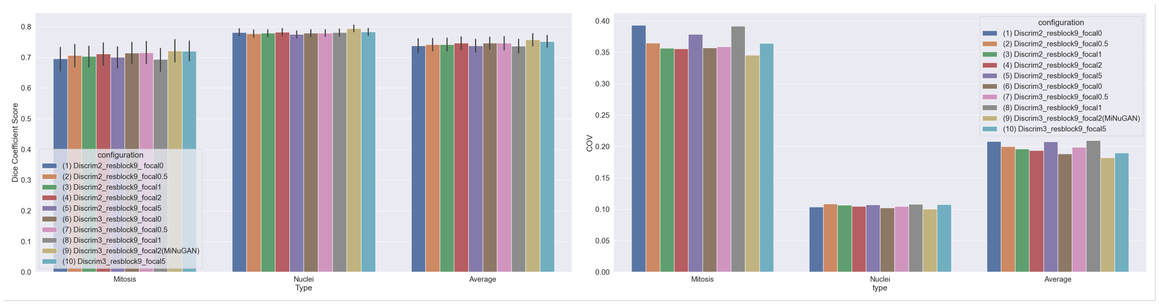

Figure 4: Mean DSC and DSC coefficient of variation (CoV) for cGAN configurations.

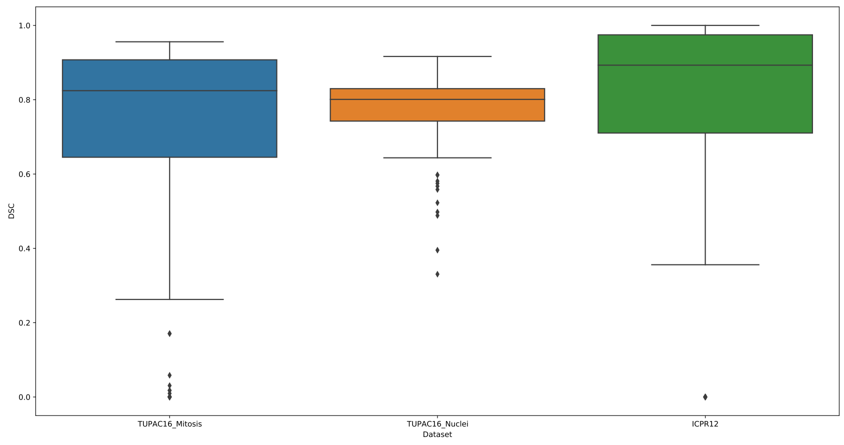

Figure 5: Mitosis segmentation performance for TUPAC16 and ICPR12.

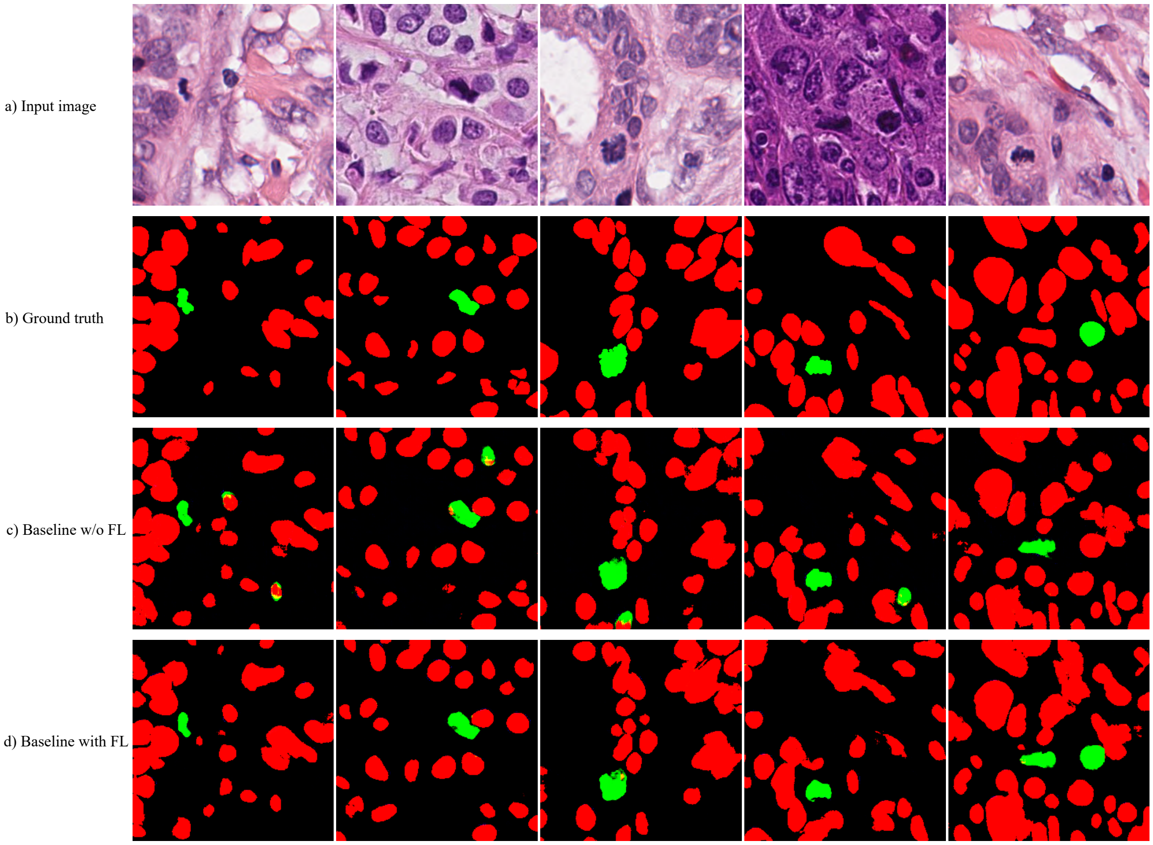

Figure 6: cGAN segmentation results with and without focal loss. a) original images, b) ground truth, c) configuration 1 (baseline without focal loss) and d) configuration 4 (baseline with focal loss).

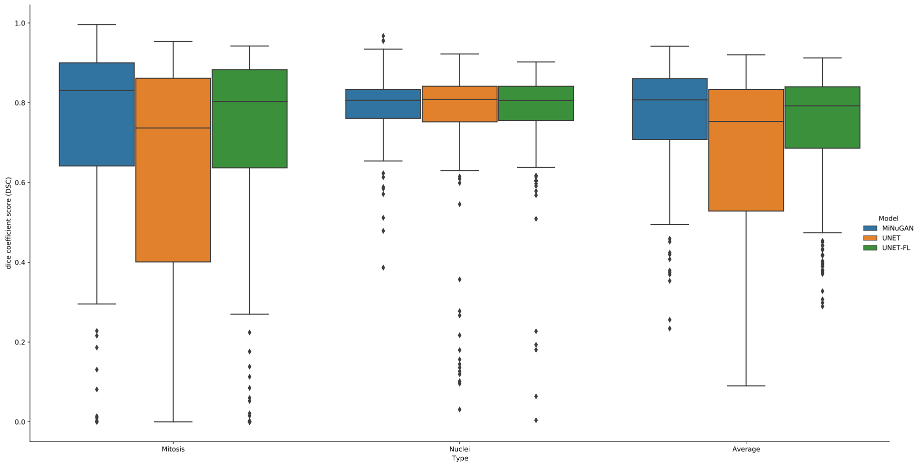

Figure 7: DSC distributions for mitoses and nuclei for MiNuGAN, UNET, UNET/focal.

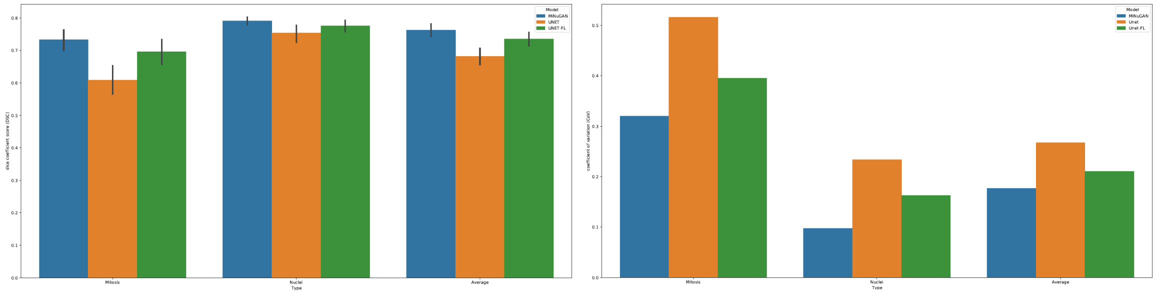

Figure 8: Mean DSC and DSC coefficient of variation for MiNuGAN, UNET, UNET/focal.

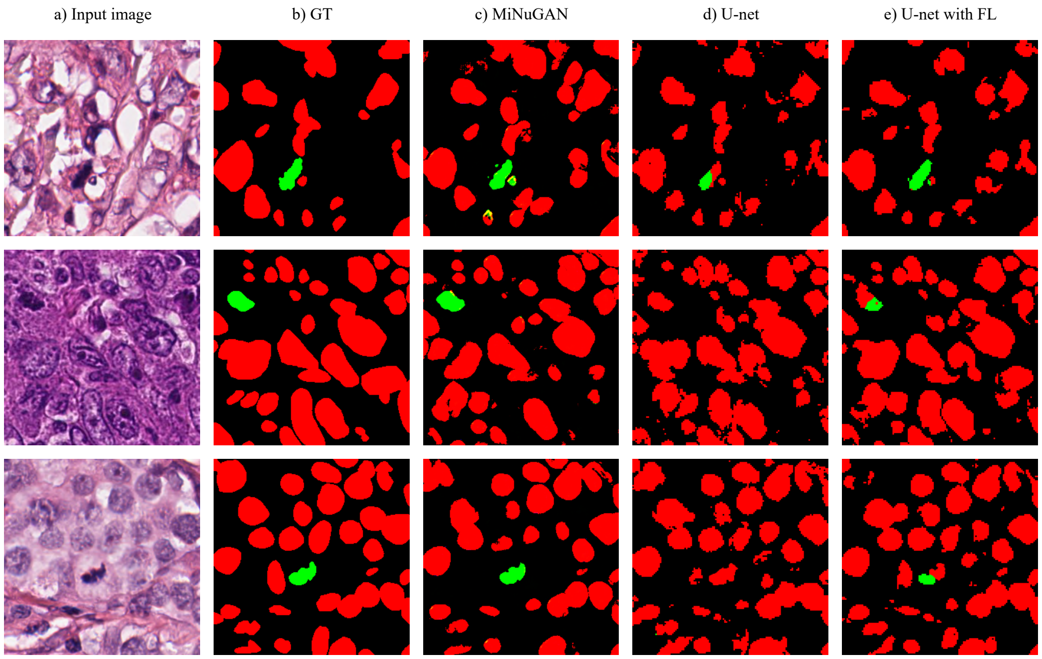

Figure 9: Segmentation results using three different models. a) input image, b) ground truth, c) MiNUGAN d) U-Net e) U-Net with focal loss.

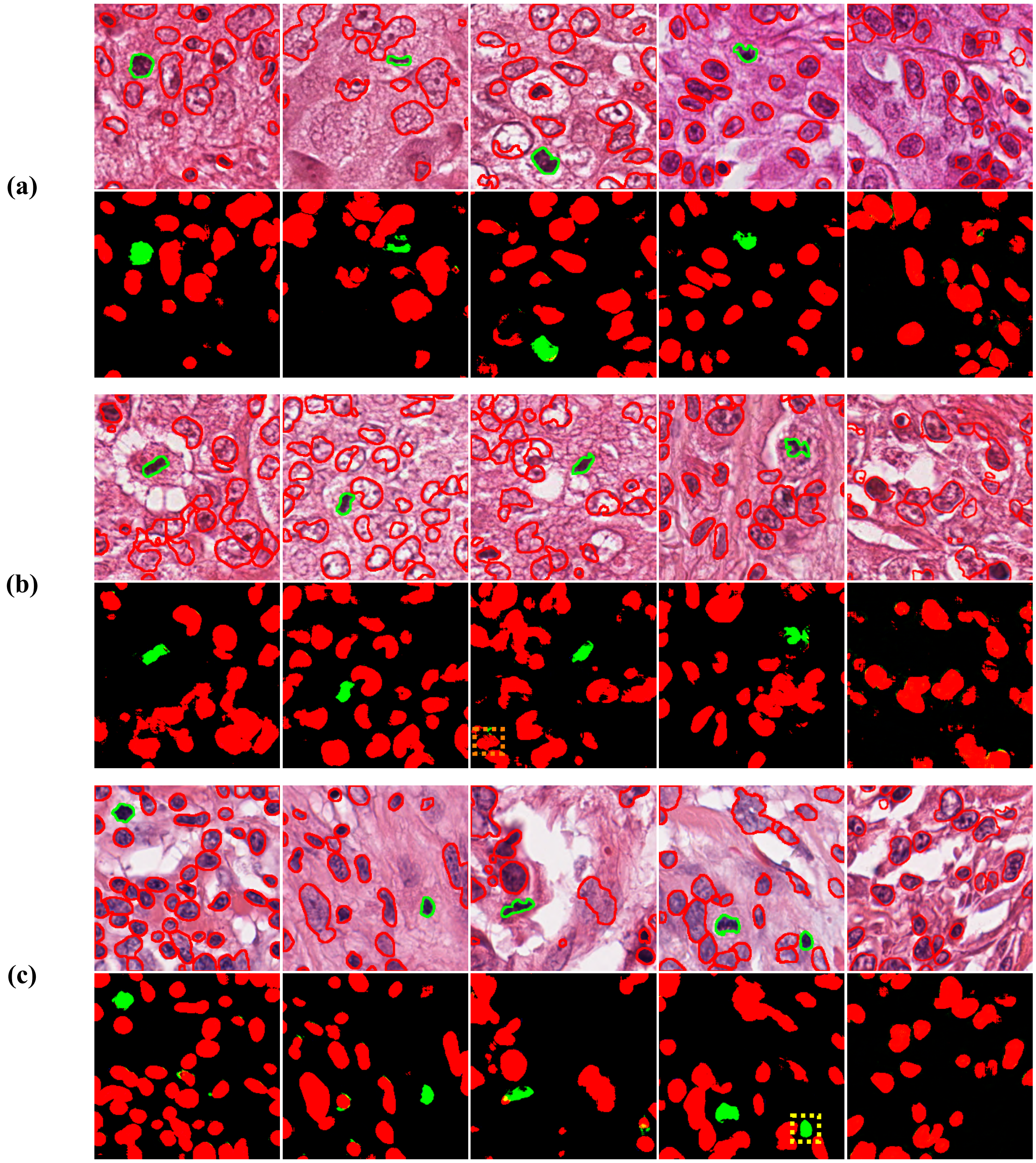

Figure 10: Segmentation and detection of mitosis using MiNuGAN for a) ICPR12, b) ICPR14, c) TUPAC16. Segmented mitoses are shown in green and nuclei in red. False positives are denoted by a yellow box, and false negatives by an orange box.
